# Comparison of the Acute Postprandial Circulating B-Vitamin and Vitamer Responses to Single Breakfast Meals in Young and Older Individuals: Preliminary Secondary Outcomes of a Randomized Controlled Trial

**DOI:** 10.3390/nu11122893

**Published:** 2019-11-28

**Authors:** Pankaja Sharma, Nicola Gillies, Shikha Pundir, Chantal A. Pileggi, James F. Markworth, Eric B. Thorstensen, David Cameron-Smith, Amber M. Milan

**Affiliations:** 1 The Liggins Institute, The University of Auckland, Auckland 1023, New Zealand; p.sharma@auckland.ac.nz (P.S.); n.gillies@auckland.ac.nz (N.G.); s.pundir@auckland.ac.nz (S.P.); c.pileggi@auckland.ac.nz (C.A.P.); jmarkwor@med.umich.edu (J.F.M.); e.thorstensen@auckland.ac.nz (E.B.T.); d.cameron-smith@auckland.ac.nz (D.C.-S.); 2 The Riddet Institute, Palmerston North 4442, New Zealand; 3 Food & Bio-based Products Group, AgResearch Grasslands, Palmerston North 4442, New Zealand

**Keywords:** ageing, B-vitamins, realistic meals, food composition, postprandial response, UHPLC-MS/MS

## Abstract

B-vitamin deficiency is common in ageing populations either due to altered dietary habits or altered digestive and metabolic functions. There is limited data on the acute circulating concentrations of B-vitamins and their various forms (vitamers), following ingestion of realistic meals. This study compared the acute circulating B-vitamin and vitamer responses to either an energy-dense (ED) or a nutrient-dense (ND) breakfast meal, consumed in a randomized cross-over sequence, in older and younger adults (*n* = 15 and 15, aged 67.3 ± 1.5 and 22.7 ± 0.5 years (mean ± SEM), respectively). Eleven differing B-vitamins and vitamers were determined in plasma samples by ultra-high-performance liquid chromatography-tandem mass spectrometry, in the fasting and postprandial state (hourly for 5 h). While postprandial thiamine concentration increased following both meals, riboflavin increased only following a ND meal in both age groups. Many vitamins including nicotinic acid, pantothenic acid, pyridoxal, pyridoxamine, pyridoxal-5’phosphate, and 4-pyridoxic acid remained unaltered, and flavin mononucleotide (FMN), nicotinamide and nicotinuric acid concentrations reduced following both meals. Biological age and food composition had minimal impact on postprandial B-vitamin concentrations, yet the differences between the ED and ND meals for riboflavin highlight the importance of riboflavin intake to achieve adequacy.

## 1. Introduction

B-vitamin deficiency is common in ageing populations [1,2], with implications for common co-morbidities of ageing such as cognitive function [3], cardiovascular diseases and inflammation [4]. It is assumed that micronutrient deficiencies are primarily the consequence of dietary inadequacy, compounded by poor diet quality [5], although altered post-meal digestion and reduced postprandial absorption may also contribute [6]. Unlike fat-soluble vitamins [7,8] and minerals [9] that are also accumulated in body stores [9,10], water-soluble B-vitamins are transiently available in small quantities from the diet [11]. However, the effective absorption and ultimate biological functions of B-vitamins may be impacted both by their bioaccessibility, the ability of B-vitamins to be released from the food matrix, as well as the gastrointestinal function. The B-vitamins require complex digestive processing [12] and subsequent metabolic conversion into the active chemical forms, termed vitamers, that act as co-factors in diverse cellular functions [13]. However, despite the importance as coenzymes [14] and the transient nature of B-vitamins in circulation [13], there remain limited descriptions of the postprandial dynamics of B-group vitamin absorption and metabolism.

The post-meal availability of B-vitamins may be impacted by factors influencing digestion and metabolism, including age and meal composition. Ageing impacts postprandial responses, seen as hypertriglyceridaemia [15] and delayed absorption of amino acids [16]. Currently, out of the few published bioavailability studies, only vitamin B_6_ [15,17] and B_12_ [18] have been reported in ageing, despite the known prevalence of impaired vitamin B_12_ absorption in older adults [19]. These studies found no age effect on postprandial B_6_ response [17], 24-h urinary vitamin B_6_ excretion [15] and fasting vitamin B_12_ [18] concentrations following ingestion of synthetic vitamin B_6_ [15,17] and vitamin B_12_ [18] doses, but not real meals. Comparisons between complex meal types, which are known to differently influence postprandial metabolic responses [20], in young adults have found a minimal impact of food form on vitamin B_6_, demonstrating similarity between fortified apple juice vs. ground beef [21] or vitamin water and mixed meals [22]; however, post-meal B-vitamin absorption in older adults is largely unknown. Better understanding the impact of food composition on B-vitamin bioavailability may provide insight into the impact of diet quality and food choices on achieving micronutrient adequacy for the elderly.

Previous studies of B-vitamin bioavailability have typically focused on isolated vitamins, such as vitamin B_6_ [15,17,22], vitamin B_12_ [22,23], riboflavin, and folate [23], which may contribute to the minimal reporting of the effects of increasing age or meal composition on bioavailability. These targeted studies have provided little additional knowledge of the closely-related vitamers. Indeed, the postprandial bioavailability of thiamine, pantothenic acid, riboflavin, and niacin has largely been overlooked. These and their vitamers can be detected using the most current targeted metabolomics techniques [24] to provide a comprehensive profile with a single run, with the exception of large molecular mass vitamins such as B_12_. These neglected but active vitamers undergo interrelated conversion, impacting circulating concentrations. Flavin mononucleotide (FMN) is the phosphorylated active co-enzyme form of riboflavin [25] which enters circulation along with riboflavin. Similarly, vitamin B_6_ exists as multiple vitamer forms: pyridoxine; pyridoxal; pyridoxamine, their phosphorylated forms, including the active coenzyme form pyridoxal-5 phosphate (PLP); and the excretory metabolite 4-pyridoxic acid (4-PA) [26]. Likewise, nicotinic acid (niacin) and nicotinamide (vitamin B_3_ vitamers) are precursors of the active coenzymes nicotinamide adenine dinucleotide (NAD) or the phosphorylated NADP, and their metabolic end-product nicotinuric acid. With their diverse and important roles as coenzymes [14], the postprandial availability of these vitamers can provide insight into their acute absorption and conversion, and the subtle impact of factors such as age or meal composition on bioavailability.

Therefore, this study profiled the postprandial circulating B-vitamin and their vitamer responses to single breakfast meals, in healthy older and younger individuals. This study also examined whether the circulating status of these vitamins and vitamers differed after acute ingestion of meals with differing food and nutrient compositions. For this, contrasting meals were selected to represent opposing diet qualities; an energy-dense (ED) refined, high fat meal, and a nutrient-dense (ND) minimally-refined low fat meal. It was hypothesized that the acute B-vitamin response to a single meal would be attenuated with ageing and that the food composition of a non-formulated ED or ND meal would have a different impact on the postprandial circulating status of these vitamins and vitamers.

## 2. Materials and Methods

### 2.1. Study Design

The study was a randomized cross-over clinical trial conducted between October 2012 and July 2013 at the Clinical Research Unit located at the Liggins Institute, University of Auckland, Auckland, New Zealand. It was designed to assess the acute metabolic and inflammatory responses to a low-fat or a high-fat breakfast meal in older people [27]. This study was conducted according to the guidelines laid down in the Declaration of Helsinki and all procedures involving human subjects were approved by the University of Auckland Human Participants and Ethics Committee (reference no. 8026). Written informed consent was obtained from all subjects. This study was also registered at Australian New Zealand Clinical Trials Registry at anzctr.org.au (ID: ACTRN12612000515897).

The details of the study design, CONSORT participant flow diagram and primary outcomes have been described elsewhere [27,28]. In brief, 30 healthy (BMI 18–30 kg/m^2^) younger (20–25 years old, *n* = 15) and older (60–75 years old, *n* = 15) participants were recruited through local newspaper advertisements to consume either a high-fat or low-fat meal in a cross-over design. The composition of the high fat meal had a high energy density and low nutrient density (energy dense meal; ED), while the low-fat meal had a low energy density and high nutrient density (nutrient dense meal; ND). Initially, the age range of eligible older adults was 70–75 years, which was amended due to difficulty in recruiting eligible older adults above 70 years of age. Participants having a history of cardiovascular or metabolic diseases, abnormal metabolism and taking medications (anti-inflammatory drugs and statins) that would interfere with the primary outcome of the study were excluded. However, individuals taking antihypertensive, diuretics, antidepressants, and vitamin D and calcium supplements were included in the study. Eligible participants attended two clinical visits, with a wash out period of a minimum of 2 weeks between interventions, to consume the meals in a random sequence generated using www.random.org. The random sequence of meal allocations was concealed in sealed envelopes prior to the first intervention visit.

### 2.2. Test Meals

The ED breakfast was a standard test meal implemented in other studies [29] and purchased from McDonald’s Restaurant, and the ND meal was prepared onsite by the researchers [27]. The ED meal comprised of an English muffin, egg, sausage patty, cheese slice, and hash brown with a total energy density of 2.3 kcal/g and a nutrient density score of 24.9% DV/kcal/g (Supplemental Table S1). The ND meal consisted of rolled oats, cottage cheese, wholemeal and grain bread, reduced fat peanut butter, fresh peach, and trim milk with a total energy density of 0.9 kcal/g and a nutrient density score of 67.7% DV/kcal/g. Both meals contained 49.8 g protein and 77.4 g carbohydrate comprising of 9.4 g dietary fiber in the ED meal and 13.2 g in the ND meal. Total energy was 1108 kcal (4530 kJ) and 685 kcal (2790 kJ) in the ED and ND meals, respectively. The B-vitamin content of the test meals are presented in Table 1.

The intake of B-vitamins from ED and ND meal ingredients was estimated using the nutritional analysis software Foodworks 8 Professional (Xyris Software PTY LTD, Brisbane, Australia) using the New Zealand Food Composition Database (FOODfiles™ 2016 Version 1).

The nutrient density of each meal (Supplemental Table S1) was calculated based on previously published methods [30] which define nutrient density of single foods or meals, as opposed to other methods which define nutrient density in terms of daily dietary intake [31]. Briefly, a nutrient adequacy score was calculated for 15 of 16 defined nutrients; pantothenic acid was not included as this nutrient is unavailable in the New Zealand Food Composition Database for some meal items (Table 1). The nutrient adequacy score for each diet was calculated as the mean of percent daily values (DV) [30] for those 15 nutrients (Equation (1)). The nutrient adequacy score for each diet was then divided by the diet’s energy density (Equation (2)) to define the nutrient density of each diet (Equation (3)). Energy density is expressed as energy provided relative to the weight of the meal (kcal/g; Supplemental Table S1). The mathematical expressions used for calculations are as follows, 

nutrient adequacy score = (∑ (nutrient intake/DV_2000 kcal_) × 100)/15 (1)

energy density = meal energy (kcal)/meal weight (g)(2)

nutrient density = nutrient adequacy score/energy density(3)

### 2.3. Study Procedures

Participants arrived fasted on both the intervention days and avoided high-fat food, anti-inflammatory medications, supplements, and vigorous physical activity the day before each visit. Participants were instructed to eat all the test meal items within 15 min. Water was provided ad libitum, not exceeding a total of 500 mL throughout the 5 h postprandial period. Anthropometric data and baseline blood samples (EDTA) were collected before the ingestion of the test breakfast meals by the participants in a randomized sequence. Subsequent blood samples were collected hourly after the meal until 5 h. The blood samples were centrifuged to obtain plasma which was stored at −80 °C until analysis.

### 2.4. Biochemical Measures

Plasma glucose, total cholesterol, low-density lipoprotein cholesterol (LDL-C), high-density lipoprotein cholesterol (HDL-C), total cholesterol, and triglycerides (TG) were measured by enzymatic colorimetric assay using Hitachi 902 autoanalyser (Hitachi High Technologies Corporation, Tokyo, Japan). Plasma insulin was measured using an Abbott AxSYM system (Abbott Laboratories, Abbott Park, IL, USA) by microparticle enzyme immunoassay.

### 2.5. Analysis of B-Vitamins in Plasma Samples

The analysis of plasma B-vitamins including thiamine; riboflavin and its vitamer FMN; the B_3_ vitamers: nicotinic acid, nicotinamide and nicotinuric acid, and pantothenic acid; and the B_6_ vitamers pyridoxal, pyridoxamine, PLP, and 4-pyridoxic acid were performed by ultra-high-performance liquid chromatography-tandem mass spectrometry (UHPLC-MS/MS) using a method published previously [28], with slight modifications, as detailed below.

#### 2.5.1. Sample Preparation 

An Eppendorf epMotion® 5075 automated liquid handling system (Eppendorf, AG, Hamburg, Germany) was programmed using integrated PC with epBlue software (version 10.09.0000, Eppendorf AG, Hamburg, Germany), for plasma sample preparation before analysis of B-vitamins using UHPLC-MS/MS. A 10-point matrix-matched standard calibration using a solution of 4% BSA in 0.1 M PBS was constructed at varying concentration ranges. For protein precipitation, 400 µL of methanol containing 0.3% acetic acid and 2.5% H_2_O was pipetted into a 2 mL square 96-well Impact^TM^ protein precipitation filter plate (Phenomenex, Torrance, CA, USA), followed by the addition of 100 µL of standard, quality control (QCs) or plasma sample. All the wells with samples, standards, QCs and a blank with BSA were spiked with 10 µL of internal standard mix except for a second blank with H_2_O. The samples were mixed for 5 min using the thermomixer component of the robot. The precipitated samples were filtered into a 96-well square collection plate by applying a vacuum of 280 mbar for 5 min. The top plate containing the protein precipitate was discarded and the bottom plate containing the filtered extract was placed in speed-vac concentrator for 3 h (Savant SC250EXP-230 SpeedVac^TM^, Thermo Scientific, Asheville, NC, USA) to evaporate the protein precipitation solvent. The concentrated samples were reconstituted with 200 µL of the reconstitution solvent made up of water containing 5% acetic acid, 0.2% heptafluorobutyric acid and 1% of ascorbic acid. The plate was covered using a square 96-well silicone lid and placed in the autosampler of the UHPLC-MS/MS for analysis.

#### 2.5.2. Liquid Chromatography and Mass Spectrometry

The separation of B-vitamins and vitamers was performed using a gradient of increasing acetonitrile over a 14 min run time using a reversed-phase Kinetex 2.6 µm F5 100Å LC column 150 × 2.1 mm (Phenomenex, Torrance, CA, USA) on a Vanquish™ UHPLC System (Thermo Fisher Scientific, USA) at a constant flow rate of 0.2 mL/min; the injection volume was 10 µL. Water containing 5% acetic acid and 0.2% HFBA was used as mobile phase A and mobile phase B was acetonitrile. Mass spectrometry was conducted using TSQ Quantiva (Thermo Fisher Scientific) system in positive electrospray ionization (H-ESI) mode. Spray voltage was set to 4000 V, sheath gas 40, auxiliary gas 10, and sweep gas at 1 (all arbitrary units). The ion transfer tube temperature and vaporizer temperature were set at 350·°C. Data was generated by the integrated Thermo Xcalibur™ mass spectrometry data system software (Thermo Xcalibur version 4.1, Thermo Fisher Scientific Inc. US).

### 2.6. Data Interpretation and Statistical Analysis

Sample size was based on the primary outcome of elevated postprandial lipemia as have been reported earlier [32], with 15 subjects per group. Very few data are available to base expected power for postprandial differences in all the B-vitamin and vitamer concentrations between old and young adults or different meal types. Moreover, individual vitamins and vitamers have a wide range of responsiveness, so it is unlikely that this study is powered sufficiently to capture group or meal effects for all measured vitamins and vitamers, many of which have not yet been described post-ingestion. Therefore, plasma PLP, the B_6_-vitamer with a wide range of functions as a coenzyme, and riboflavin, being present in considerable amounts in both the test meals in different foods (eggs vs. milk), were selected for sample size calculations. To provide 80% power with alpha set at 5%, based on previously reported baseline differences between old and young subjects [16], six subjects per group would be required to detect a meaningful 44% reduction in mean PLP concentrations from 76 nmol/L to 42 nmol/L with a standard deviation (SD) of 21. Similarly, to compare the effect of meal on B-vitamin concentrations, 16 subjects per group were calculated to detect a difference of 0.2 nmol/L with a SD of 0.2 in postprandial mean riboflavin concentrations of 1.5 following milk and 1.3 nmol/L following spinach [33]. 

Data are presented as means ± standard error of means (SEM). The incremental area under the curve (iAUC) values was obtained after correction for baseline values. The homoeostatic model assessment of insulin resistance (HOMA-IR) was calculated based on the fasting glucose and insulin concentrations using the equation published in the literature [34]. Statistical significance was tested using generalized linear mixed models (IBM SPSS Statistics 25, IBM Corp., Armonk, NY, USA), using the syntax model which allows pairwise missing data, with meal and time compared within-subject and age compared as a between-subject factor. Sex was not included as a factor due to the small sample size. Post hoc testing using Sidak-Holm adjustments for all pairwise comparisons and baseline measures and iAUC were compared using Student’s *t* test. Alpha was set at *p* < 0.05.

## 3. Results

### 3.1. Baseline Characteristics

The baseline characteristics (Table 2) of study participants have previously been detailed elsewhere [32]. The age of older (67.3 ± 1.5 years) and younger (22.7 ± 0.5 years) subjects (*n* = 15 in each age group, with seven females in the younger and nine females in the older group) was significantly different (*p* < 0.001) and the BMI was not different (*p* = 0.480) between older (24.4 ± 1.03 kg/m^2^) and younger (23.7 ± 0.80 kg/m^2^) subjects. The fasting markers of insulin sensitivity including glucose, insulin and HOMA-IR were not different between the older and younger subjects. LDL, HDL and total cholesterol were significantly higher in older compared to younger subjects, whereas TAG was not different between age groups.

### 3.2. Baseline Vitamin Status

The older subjects had significantly higher baseline concentrations of nicotinic acid (*p* = 0.001) and riboflavin (*p* = 0.015; Table 3) compared to the younger counterparts. The baseline concentrations for all other vitamins and vitamers, including pantothenic acid (*p* = 0.054), did not differ significantly between the two age groups (*p* > 0.05). 

### 3.3. Vitamins Present in the Meals

The nutrient density of the ND meal was 2.7-fold higher than the ED meal, which had a 2.6-fold higher energy density (Supplemental Table S1). The two meals differed in quantities of several water-soluble vitamins (Table 1). The thiamine, niacin and pantothenic acid contents of the ED meal were higher than the ND meal, vitamin B6 was similar, whereas, riboflavin was higher in the ND meal. The thiamine content of the ND meal was 4.6 times lower than the ED meal. Similarly, niacin in the ED meal was 1.4 mg greater than the ND meal. Pantothenic acid in the ED meal was 11.7-fold greater than the ND meal. In contrast, riboflavin in the ND meal was 1.8-fold higher than the ED meal. 

### 3.4. Vitamin Responses to Meal Composition

**Figure 1:**
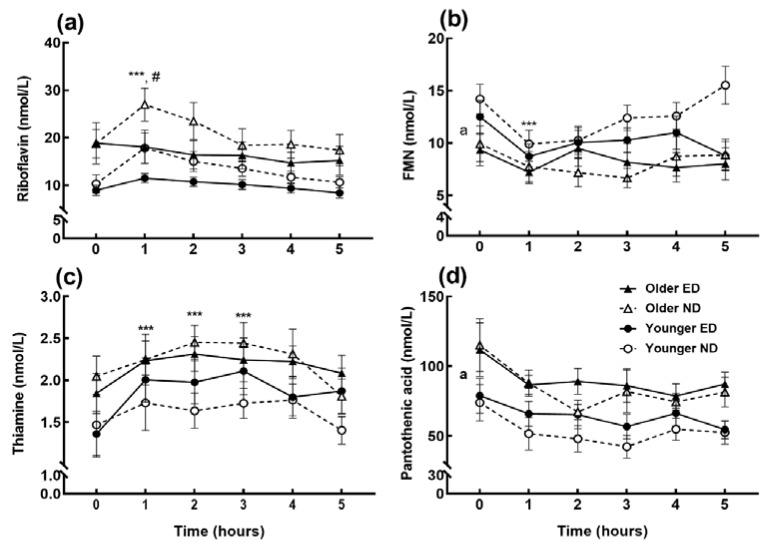
(**a**) Postprandial circulating riboflavin; (**b**) the vitamer flavin mononucleotide (FMN); (**c**) thiamine; and (**d**) pantothenic acid concentrations (nmol/L) in energy-dense (ED) and nutrient-dense (ND) breakfast fed elderly and young subjects at baseline (0) and hourly for 5 h. Data points and error bars represent means and ±SEM respectively, *n* = 15 in each age group. A meal x time interaction (*p* = 0.002) showed increased riboflavin concentrations from baseline at 1 h post-ND meal compared to baseline (***, *p* < 0.001); significant difference between ED and ND meal treatments at 1 h (#, *p* = 0.008). There was a main age effect (a, *p* = 0.008) and main time effect (***, *p* < 0.001) for FMN. There was a main time effect (*p* = 0.001) in thiamine and main age effect (a, *p* < 0.001) for pantothenic acid.

Regardless of age, only acute ingestion of the ND meal with greater riboflavin content increased postprandial riboflavin concentrations (meal × time interaction, *p* = 0.002). At 1 h following the ND meal, plasma riboflavin concentrations were higher compared to baseline (*p* < 0.001; Figure 1a) and compared to the ED meal (*p* = 0.008) containing 54% less riboflavin. This difference also corresponded with a higher riboflavin iAUC after the ND meal (*p* = 0.012) which was independent of age (age x meal interaction *p* = 0.245, age effect *p* = 0.058; Table 4). However, the postprandial concentration of the riboflavin vitamer flavin mononucleotide (FMN) reduced regardless of age or meal (time effect *p* < 0.001; Figure 1b), also shown by the negative FMN iAUC, which was lower in the older subjects (age effect *p* = 0.008).

**Figure 2:**
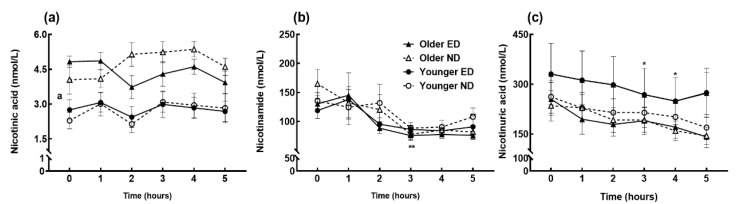
(**a**) Postprandial circulating concentrations of nicotinic acid; (**b**) nicotinamide; and (**c**) nicotinuric acid in energy-dense (ED) and nutrient-dense (ND) breakfast meal fed to older and younger subjects at baseline (0) and hourly until 5 h. Data points and error bars represent means and ±SEM respectively, *n* = 15 in each age group. No interactions were present between any independent factors (age, meal and time). There was a main time effect in nicotinamide (**, *p* < 0.01) and nicotinuric acid (*, *p* < 0.05) and a main age effect (a, *p* < 0.001) in nicotinic acid concentrations.

**Figure 3:**
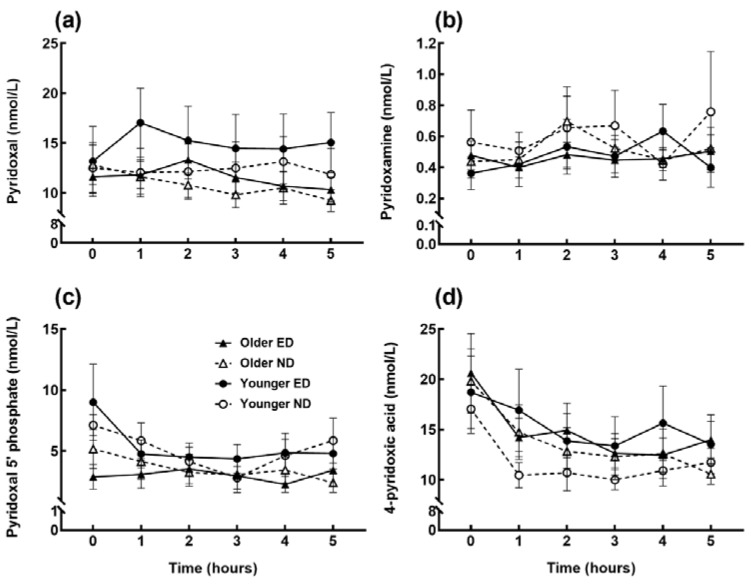
Postprandial circulating concentrations of the vitamers of vitamin B_6_, (**a**) pyridoxal; (**b**) pyridoxamine; (**c**) pyridoxal 5’-phosphate (PLP); and (**d**) 4-pyridoxic acid (4-PA) in energy-dense (ED) and nutrient-dense (ND) breakfast meal fed to older and younger subjects at baseline (0) and hourly until 5 h. Data points and error bars represent means and ± SEM respectively, *n* = 15 in each age group. No significant changes were found in any of the B_6_ vitamers.

Similarly, postprandial changes in circulating thiamine (Figure 1c), pantothenic acid (Figure 1d), and all measured concentrations of the vitamers of niacin (Figure 2) and B_6_ (Figure 3) were independent of age or meal. Thiamine concentrations at 1–3 h increased compared to baseline (*p* < 0.01, time effect *p* = 0.001), whereas the B_3_ vitamers nicotinamide and nicotinuric acid decreased (time effect *p* = 0.001, *p* < 0.01 at 3–5 h and *p* = 0.001, *p* < 0.05 at 4–5 h respectively). Older subjects had higher concentrations of nicotinic acid, another B_3_ vitamer, and pantothenic acid (age effect *p* < 0.001) compared to the younger group. Whilst the B_6_ vitamers did not change after meal ingestion, the overall pyridoxal iAUC (Table 4) was greater after the ED meal (meal effect *p* = 0.025) and in younger subjects (age effect *p* = 0.015), although with no age x meal interaction (*p* = 0.938). 

## 4. Discussion

Despite extensive evidence describing the prevalence and impact of micronutrient inadequacy in the elderly, the post-meal responsiveness of the diverse members of the B-vitamin family in older people has been overlooked. This study examined the acute response of B-vitamins to a single meal with varied food composition in both younger and older adults through comprehensive mass spectrometry profiling of vitamins and associated vitamers. Despite age differences in fasting circulating B-vitamin and vitamer concentrations, we did not observe differences in post-meal concentrations between older and younger subjects, contrary to our hypothesis that the acute response of B-vitamins would be impacted by ageing. Yet, regardless of age, this study demonstrates that meal composition impacts postprandial concentrations of riboflavin, with minimal impact on other B-vitamins. The rapid postprandial rise in riboflavin following the single ND meal was likely reflective of both greater riboflavin content and differing food sources and structures. This difference in riboflavin bioavailability between meals demonstrates that the ingestion of food with a lower vitamin content and differing meal compositions may impair postprandial availability of only specific vitamins, with possible implications for the efficacy of these ingested vitamins. Hence, food form, in addition to total dietary intake, may be an important consideration for predicting certain circulating vitamin or vitamer status and availability, highlighting the importance of concurrent analysis of circulating vitamers when assessing vitamin status.

Both older and younger subjects, in this study, had similar postprandial vitamin and vitamer responses, indicating that the postprandial availability of these vitamins from food does not vary markedly with age. This finding agrees with previous comparisons of vitamin B_6_ bioavailability in healthy elderly men [15,17] following vitamin B_6_ ingestion in circulation [17] and excretion [15], although these were in response to supplements in higher doses unlike the present study. Such a lack of difference between old and young aligns with the notion that B-vitamin malabsorption reported particularly with vitamin B_12_ [35] in older people is typically attributable to medication use or underlying gastrointestinal or renal diseases, and not a normal process of ageing [35]. Therefore, the similar response between the age groups is likely due to the healthy gastrointestinal and metabolic status of the older participants in this study, which aimed to eliminate confounding factors, but may be less representative of gastrointestinal [36] or metabolic dysfunction [36] incidence reported in available literature. Despite evidence that older people are at a greater risk of micronutrient deficiency [37], the current study suggests that the altered postprandial response of these micronutrients from complex meals with natural vitamin content is unlikely to be a key factor contributing to this elevated deficiency risk in healthy elderly. Indeed, contrary to reports of insufficient vitamin intake in older adults [38,39,40,41], fasting circulating concentration differences between older and younger adults were negligible, indicating that dietary intakes were possibly adequate in this healthy population. Inadequate intakes depleting circulating concentrations, as may be more apparent in other subsets of elderly individuals, may be expected to influence these postprandial responses, as tissue and plasma concentrations partly control intestinal absorption [12]. Deficiency of thiamine, riboflavin, pyridoxine, biotin, and folate have been reported to up-regulate intestinal uptake, whereas over-supplementation of biotin and riboflavin down-regulates the of these vitamins [12]; however, it is unclear what impact this would have on postprandial concentrations. As turnover of total B-vitamin body stores due to prolonged inadequacy may vary from years (vitamin B12), months (niacin [42]), weeks (thiamine [43,44], pantothenic acid, or B6 vitamers [45]), or even days (riboflavin, stored as FMN or FAD [46]), it is possible that postprandial absorption may be variable depending on current intake or status. However, limited comparative evidence is available to determine the expected influence of B-vitamin tissue stores on postprandial bioavailability. Yet the current study is unable to differentiate between possible age-related influences of basal intake or status, postprandial intestinal absorption, metabolic clearance, and usage of these vitamins. For this, more complex mapping of the various intermediary metabolites, with possible tissue sample analysis, is required to generate further insight into age-related alterations that may be undetectable through assessment of circulating vitamin and vitamer concentrations. 

Irrespective of participants’ age, this study showed that plasma riboflavin concentrations were elevated following the ingestion of the ND meal but not following the ED meal. Although the riboflavin content varied between the meals, food structure also differed, which may have impacted riboflavin digestion and bioaccessibility [47]. Milk, the main source of riboflavin in the ND meal, contains largely free riboflavin. This is more efficiently absorbed than the phosphorylated and active vitamer forms, flavin adenine dinucleotide (FAD) or FMN [48]. Interestingly, the postprandial concentrations of FMN did not differ with meal types in this study, which may be acceptable as only a 1.2–1.5 fold increase in plasma FMN concentration has been reported following an oral riboflavin dose as high as 20 mg previously [49]. Yet, the free riboflavin in the ED meal from cheese and eggs, which still provided a substantial 66% of the recommended dietary intake (RDI) [50], did not raise postprandial riboflavin concentrations. Water-soluble vitamins may be more bioaccessible depending on their food matrix [43]; for instance, lipo-soluble vitamins have enhanced bioaccessibility in liquid matrices (i.e., softgel capsules) [44]. Aside from structure and composition differences, the ND breakfast provided 1.8 times more riboflavin (0.79 mg) than the ED meal, which may have been the primary factor influencing postprandial concentrations. The higher total energy from the ED meal may have impacted postprandial responses, such as slower gastric emptying [45] or greater insulin responses [42], which may in turn influence postprandial absorption of B-vitamins, although a previous postprandial comparison of B-vitamin availability from calorically unmatched meals demonstrated no difference [22]. Hence, while this study aimed to compare responses to two different meal compositions, the variation in vitamin content, particularly riboflavin, likely influenced postprandial responses, making it difficult to determine the effects of food composition alone, although niacin and vitamin B_6_ intakes were comparable between meals.

Many of the B-vitamins and vitamers did not increase in postprandial circulation, in the present study, despite the meals containing appreciable quantities similar to or exceeding the RDIs. This included pantothenic acid, niacin vitamers (nicotinic acid, nicotinamide, nicotinuric acid), and B_6_ vitamers (pyridoxal, pyridoxamine, PLP and 4-PA). Previous studies have demonstrated increased circulating concentrations after thiamine doses of 50 [46] or 100 mg [51]; a riboflavin dose of 20–40 mg [49]; a niacin dose of 500 mg [52]; or a pyridoxine hydrochloride dose of 10 mg (raising plasma pyridoxal, PLP and 4-PA concentrations) [53]. These doses were all well in excess of typical meals, including those in the current study. As such, the relatively low doses provided, such as thiamine, could account for the lack of postprandial difference between the ED and ND meal, despite compositional differences in the meals. This discrepancy may arise due to the differential absorption mechanisms (active transport vs passive diffusion) dependent on relative amounts consumed, as demonstrated in pharmacokinetic studies [46,49,51,52,53]. Indeed, there has been very limited research to date on the postprandial dynamics of these B-vitamins from realistic meals, thus it is not possible to fully explain these findings. Of the other available data, the postprandial responses of vitamin B_6_ [15,17], vitamin B_12_ and folate are described from synthetic forms used in supplements, or with meal fortification [23,43] or in formulated meals [22]. All have provided vitamin levels well in excess of the current study and well in excess of the RDI. For instance, in contrast to our findings, in formulated meals vitamin, B_6_ has been shown to be increased post-ingestion [22]; yet, this was in response to a fortified drink and simple meal with limited fiber and fat content. Unfortunately, vitamin B_12_, although previously studied [22,23], could not be measured in this study as it was not possible to include in the same mass spectrometry panel owing to the high molecular weight of this vitamin. Given the current findings which contrast with previous reports of postprandial B-vitamin responsiveness, future studies should give greater attention to their acute bioavailability from realistic meals that consider the impact of food forms and processing. 

The implications of altered postprandial B-vitamin dynamics, particularly in individuals with sub-clinical deficiencies of riboflavin or thiamine, on long-term health outcomes is not well understood from the available literature [13,54,55]. Acute effects of some of the B-vitamins at mega doses have been identified [13]. These include improved vasodilation within 2 h after a single high folic acid dose in individuals with endothelial dysfunction [56]; increased serotonin synthesis following intravenous dose of pyridoxine in an animal model [57]; and improved brain activity following a single multivitamin dose within 3 h in children [58] or 30 min in young adults [59]. However, acute examples are limited, and exhaustive B-vitamin and vitamer dynamics are unavailable. Health outcome benefits have been demonstrated with greater long term intake in compromised populations; for instance, greater riboflavin and thiamine intake have been shown to improve circulating riboflavin [55,60,61], thiamine status [62], glucose tolerance [63], general health and wellbeing [62,64], and cognitive function [65]. Despite this evidence, the role of postprandial responses in the relationship between intake and health outcomes is not yet well understood.

The selection of healthy older adults limits the generalizability of these findings to all elderly populations, for whom co-morbidities may be prevalent; however, this trade-off aimed to eliminate confounding factors that would have also impacted digestive and metabolic function. Although this study aimed to investigate the impact of meal structure on postprandial vitamin concentrations, this was limited by the variability in vitamin content between the meals. However, designing comparator meals that differ in food structure but are identical in content across a range of vitamins would not only be challenging, but would also likely require the use of contrived meal formulations that will ultimately be poorly representative of realistic meals. The selection of breakfast style foods used in this study may have also under-represented specific water-soluble vitamins found in non-breakfast foods consumed at other meal times. With multiple vitamin endpoints, and as a secondary outcome, the power to detect differences may also be limited by the smaller sample size, estimated for riboflavin and the B_6_-vitamer PLP. Although it was not possible to estimate the effect size for all vitamers measured, due to unavailability of data in literature, it is possible that the selected sample size precluded detection of differences in some compounds. Indeed, B-vitamin studies of similar design have similarly included small numbers of subjects (*n* = 12 to 20 [15,21,22]), warranting validation in larger populations. Nonetheless, despite the limitations, this study provides comprehensive vitamin and vitamer dynamic profiles not reported previously, providing a basis for effect size estimates to more robustly assess these in future studies.

## 5. Conclusions

This study showed minimal impact of age or meal composition in either age group on postprandial B-vitamins and related vitamers in circulation following realistic meals. This suggests that B-vitamin availability following realistic meals is not compromised in healthy older individuals. Despite limited differences in B-vitamin responses between meals, postprandial riboflavin concentrations were greater following a ND meal that differed in both overall food composition and riboflavin content than an ED meal, highlighting the importance of absolute intake required to raise plasma concentrations of these vitamins. An expanded understanding of the differential influence of intake relative to food composition, along with the influence of postprandial B-vitamin dynamics on health outcomes, is still required to inform food choices for optimal vitamin bioavailability in the elderly, although, for all ages, choosing foods rich in all B-vitamins should remain the best strategy to maintain sufficiency.

## Figures and Tables

**Table 1:** The quantity of B-vitamins present in each meal ingredient with the estimated total intakes from the meals ingested.

	Thiamine (mg)	Riboflavin (mg)	Niacin E (mg)	Vitamin B_6_ (mg)	Pantothenic Acid (mg)
ND meal items					
Peach (154 g)	0.02	0.03	1.14	0.12	0.31
Mixed grain bread (42 g)	0.06	0.06	0.93	0.01	n.d.
Peanut butter (22 g)	0.00	0.03	4.61	0.01	n.d.
Oats, toasted, rolled (45 g)	0.14	0.05	1.15	0.18	n.d.
Cow milk, 0.1% fat (300 mL)	0.06	0.94	2.71	0.09	n.d.
Cottage cheese, light, 1% fat (150 g)	0.06	0.18	3.90	0.11	n.d.
Total	0.34	1.28	14.45	0.52	0.31
ED meal items					
Cheese, sliced, reduced fat (8.4 g)	0.00	0.11	1.02	0.00	0.10
Muffin (100 g)	0.34	0.08	4.20	0.11	0.30
Breakfast sausage (110 g)	0.55	0.10	3.63	0.14	0.53
Potato, hash brown (150 g)	0.48	0.05	3.90	0.09	0.71
Egg, fried (110 g)	0.14	0.45	4.40	0.17	1.98
Total	1.51	0.79	17.15	0.51	3.62

ND, nutrient dense meal; ED, energy dense meal; Niacin E, niacin equivalents; n.d., no data available from the database. Values presented are based on data available from the New Zealand Food Composition Database (FOODfiles™ 2016 Version 1)

**Table 2:** Baseline characteristics of study participants including fasting plasma glucose, insulin and lipid profile before ingestion of the test meals.

Measures	ND		ED	
	Older	Younger	Older	Younger
LDL (mmol/L)	3.1 ± 0.2	2.5 ± 0.2	3.0 ± 0.2	2.4 ± 0.20 **
HDL (mmol/L)	1.8 ± 0.1	1.3 ± 0.0	1.8 ± 0.1	1.4 ± 0.1 ***
Cholesterol (mmol/L)	5.1 ± 0.2	4.0 ± 0.2	5.0 ± 0.2	4.0 ± 0.2 ***
TAG (mmol/L)	0.9 ± 0.1	0.8 ± 0.1	0.9 ± 0.1	0.8 ± 0.1
Glucose (mmol/L)	5.2 ± 0.2	5.1 ± 0.1	5.2 ± 0.2	5.1 ± 0.1
Insulin (mmol/L)	8.7 ± 1.4	9.5 ± 1.3	8.7 ± 1.9	9.0 ± 1.0
HOMA-IR	2.1 ± 0.4	2.2 ± 0.4	1.7 ± 0.3	2.0 ± 0.2

Values presented as means ± SEMs, *n* = 15 in each age group. ND: nutrient dense meal; ED: energy dense meal; LDL: low density lipoproteins; HDL: high density lipoprotein; TAG: triacylglycerides; HOMA-IR, homeostatic model assessment of insulin resistance. Statistical significance was determined by one-way ANOVA (Analysis of variance). Significant difference between age groups are denoted by **, *p* < 0.01; ***, *p* < 0.001.

**Table 3:** Mean baseline circulating concentrations of B-vitamins and vitamers in younger and older subjects before ingestion of test meals.

	ND		ED	
	Older	Younger	Older	Younger
Thiamine	2.1 ± 0.2	1.45 ± 0.4	1.8 ± 0.3	1.4 ± 0.3
Riboflavin	18.7 ± 2.9 *	10.31 ± 1.9	18.8 ± 4.4 *	8.9 ± 1.06
FMN	9.9 ± 1.7	14.08 ± 1.5	9.4 ± 1.6	12.5 ± 1.6
Nicotinic acid	4.0 ± 0.6 *	2.25 ± 0.4	4.8 ± 0.3 ***	2.74 ± 0.5
Nicotinamide	164.9 ± 24.7	172.62 ± 50.0	133.0 ± 14.8	118.96 ± 14.2
Nicotinuric acid	254.9 ± 49.9	229.46 ± 48.6	254.9 ± 52.3	330.72 ± 92.5
Pantothenic acid	115.1 ± 18.8	72.4 ± 13.8	111.9 ± 20.6	78.89 ± 12.5
Pyridoxal	12.8 ± 1.9	12.8 ± 2.7	11.0 ± 1.6	13.16 ± 3.5
Pyridoxamine	0.4 ± 0.1	0.6 ± 0.2	0.5 ± 0.1	0.36 ± 0.1
PLP	5.12 ± 1.6	6.9 ± 0.9	2.9 ± 1.1	9 ± 3.1
4-Pyridoxic acid	19.8 ± 3.2	16.5 ± 2.6	20.6 ± 4.2	18.7 ± 3.6

Values presented as means (nmol/L) ± SEMs, *n* = 15 in each age group. ND: nutrient dense meal; ED: energy dense meal; FMN, flavin mononucleotide PLP, Pyridoxal-5’phosphate. There was no significant differences between baseline concentrations within groups. Significant difference between the age groups on the intervention day are denoted by *, *p* < 0.05; ***, *p* < 0.001.

**Table 4:** Area under the curve (iAUC) of B-vitamins in older and younger adults after ingestion of test meals.

	ND		ED	
	Older	Younger	Older	Younger
Thiamine	69 ± 47	57 ± 66	105 ± 45	163 ± 28
Riboflavin	675 ± 360	851 ± 265 *	−578 ± 399	365 ± 101
FMN	−592 ± 261	−662 ± 363	−329 ± 317	−712 ± 180
Nicotinic acid	235 ± 169	138 ± 90	−136 ± 89	18 ± 109
Nicotinamide	−17,118 ± 5803	−7163 ± 5198	−9675 ± 3303	−5192 ± 4469
Nicotinuric acid	−20,585 ± 5638	−12,810 ± 4719	−14,155 ± 3724	−13,423 ± 5421
Pantothenic acid	−9935 ± 4219	−6585 ± 2384	−7156 ± 5081	−4391 ± 1729
Pyridoxal	−626 ± 2726	−31 ± 331	11 ± 142	564 ± 172 *^,#^
Pyridoxamine	24 ± 22	6 ± 29	−7 ± 18	38 ± 20
PLP	−490 ± 424	−702 ± 134	37 ± 119	−1177 ± 670
4-Pyridoxic acid	−1885 ± 379	−1726 ± 517	−1897 ± 769	−1058 ± 213

Values are obtained after deducting the baseline concentrations and represent means (nmol × min/L) ± SEM, *n* = 15 in each age group. ND: nutrient-dense meal; ED: energy-dense meal; FMN, flavin mononucleotide PLP, Pyridoxal-5’phosphate. No age x meal interaction occurred. The differences due to main meal effect (*) were present for riboflavin (ND > ED, *p* = 0.012) and pyridoxal (ED > ND, *p* = 0.025) and main age effect (^#^) for pyridoxal (younger > older, *p* = 0.015).

## Supplementary Materials

The following are available online at https://www.mdpi.com/2072-6643/11/12/2893/s1, Table S1: Nutrient and energy density score of the test meals.
