# Supplementary material for: Comparison of the Acute Postprandial Circulating B-Vitamin and Vitamer Responses to Single Breakfast Meals in Young and Older Individuals: Preliminary Secondary Outcomes of a Randomized Controlled Trial
Source: Nutrients. 2019 Nov 28;11(12):2893. doi: 10.3390/nu11122893 (PMC6950174; doi:10.3390/nu11122893)
Supplement: Supplementary file 1 [file nutrients-11-02893-s001.pdf]

Supplemental Table

Sharma *et al* (2019). Comparison of the Acute Postprandial Circulating B-vitamin and Vitamer Responses to Single Breakfast Meals in Young and Older Individuals: A Randomized Controlled Trial. Nutrients.

Supplemental Table S1: Nutrient and energy density score of the test meals.

**Nutrients***	**ED meal** (%DV)	**ND meal** (% DV)
Protein	80.9	83.6
Dietary fibre	37.7	52.8
Vitamin A equivalent	34.9	20.2
Thiamine	126.3	28.0
Riboflavin	50.8	82.6
Niacin	137.2	115.6
Vitamin B6	31.0	31.6
Total folate DFE	39.5	107.8
Vitamin B12	82.5	66.7
Vitamin C	10.4	13.4
Vitamin E	19.8	26.0
Vitamin D	59.6	164.9
Calcium	47.6	90.6
Iron	56.5	29.8
Magnesium	28.0	44.8
**Nutrient adequacy (% DV)**	56.2	63.9
**Energy Density (kcal/g)**	2.3	0.9
**Nutrient density score (%DV/kcal/g)**	24.9	67.7

Nutrient and energy density scores were calculated as nutrient adequacy score to energy density ratio based on the 15 key nutrients according to the standard nutrient density score[(1)]. *Instead of 16 described in the original method[(1)], only 15 were included for the calculations, as pantothenic acid content of several food items were not available from the database. Nutrient content of the meals were obtained using the Foodworks software from New Zealand Food Composition Database (FOODfiles™ 2016 V.1). DV, daily value; ED, energy dense; ND, nutrient dense; DFE, Dietary folate equivalent; kcal, kilo calorie.

References

1. Darmon N, Darmon M, Maillot M, *et al.* (2005) A nutrient density standard for vegetables and fruits: Nutrients per calorie and nutrients per unit cost. *J Am Diet Assoc* **105**, 1881–1887.
